# Effect of Fermentation Time on Molecular Structure and Physicochemical Properties of Corn Ballast Starch

**DOI:** 10.3389/fnut.2022.885662

**Published:** 2022-04-27

**Authors:** Chunhong Wei, Yunfei Ge, Shuting Zhao, Dezhi Liu, Junlan Jiliu, Yunjiao Wu, Xin Hu, Mingzhi Wei, Yifei Wang, Weihao Wang, Lidong Wang, LongKui Cao

**Affiliations:** ^1^College of Food Science, Heilongjiang Bayi Agricultural University, Daqing, China; ^2^National Coarse Cereals Engineering Research Center, Heilongjiang Bayi Agricultural University, Daqing, China; ^3^Department of Marine Food Science and Technology, Gangneung-Wonju National University, Gangneung, South Korea

**Keywords:** natural fermentation, corn starch, molecular structure, functional properties, physicochemical indicators

## Abstract

The effect of fermentation treatment on the surface morphology, crystal structure, molecular weight, chain length distribution, and physicochemical properties of corn starch was investigated using natural fermentation of corn ballast. The amylose content in corn ballast starch reduced at first after natural fermentation, then grew, following the same trend as solubility. There were certain erosion marks on the surfaces of fermented corn ballast starch granules. The crystalline structure of corn ballast starch remained the same, i.e., a typical A-type crystalline structure, at different fermentation times; however, the intensities of diffraction peaks were different. The weight-average molecular weight of starch first increased and then decreased after fermentation. The content of low-molecular-weight starch (peak 3) decreased from 25.59 to 24.7% and then increased to 25.76%, while the content of high-molecular-weight starch (peak 1) increased from 51.45 to 53.26%, and then decreased to 52.52%. The fermentation time showed a negative correlation with the viscosity of starch, and the pasting temperature first increased, and then decreased. Natural fermentation can be used as a technical means to produce corn starch products as a result of the experiments' findings, and future experiments will detect and analyze the bacterial structure of corn fermentation broth in order to better understand the molecular mechanism of natural fermentation affecting the structure and physicochemical properties of corn starch.

## Introduction

As the third-largest food and cash crop in the world, maize has been extensively grown in all major continents, with a global production of 988 tons in 2014 (1). As a food crop with balanced nutrients, maize not only provides sufficient energy to the human body, but is also rich in nutrients like linoleic acid, protein, minerals, vitamins, and lutein; therefore, it receives interest from a wide range of consumer groups (2).

Starch is the main carbohydrate in maize, with content as high as 70–80%. Thus, the molecular structure of starch and its physicochemical properties directly affect the taste quality of maize and its applications. Although maize is rich in bioactive components and nutrients (3), its coarse texture and pungent taste have limited its use. The close interactions of starch, protein, and fat in maize limit the water absorption and expansion of corn flour, leading to poor quality in processing. Hence, corn and corn flour are mainly used as a feed or industrial raw materials, resulting in a huge waste of food resources (4). Studies have shown that around 78% of corns in developed countries are used in the feed industry, whereas only around 5% of corns are directly consumed. Therefore, identifying appropriate methods to improve the processing quality of corn flour to expand its applications has become the focus of current research.

The current techniques to improve corn flour mainly include the use of chemical additives (5), physical modifications (6), and biological improvements (7). However, these methods have limitations. For instance, chemical additives like calcium salts are harmful to the human body, and physical modification techniques like extrusion and puffing are expensive. Although enzymatic hydrolysis is effective, it involves complex mechanisms. Fermentation is one of the oldest food processing methods of preparing and preserving foods and is a safe and economical way to improve the nutritional and sensorial properties of food products, is widely used in the production of bread, rice noodles, beer, vinegar, and other foods (8). Organic acids and enzymes produced by microbial metabolism in the natural fermentation worked together to boost the fermentation of raw materials; hence, the modification effect of this process was significantly better than that of the single biological modification method (9).

Fermentation changes the molecular structure, physicochemical indicators, and functional properties of starch to enhance flavor and taste, extend the shelf life, and increase the nutritional value of starch-based food products. Therefore, in this experiment, we used the traditional natural fermentation process to determine the effect of different fermentation times on the molecular structure and physicochemical properties of corn starch by scanning electron microscopy (SEM), gel permeation chromatography (GPC) with multi-angle light scattering characterization (MALS), and differential scanning calorimetry (DSC). The effect of different fermentation times on the molecular structure and physicochemical properties of maize starch was determined using SEM, GPC-MALS, and DSC to provide a theoretical basis and data support for future fermented maize products.

## Materials and Methods

### Materials and Instruments

Corn ballast were purchased from Lingbi County Zhuoyue Grain and Oil Trading Co., Ltd. Hydrochloric acid (analytical grade) and sodium oxide (analytical grade) were purchased from Tianjin Damao Chemical Reagent Factory; sodium hydroxide, hydrochloric acid, potato amylose standard, acetone, and ethanol were purchased from Sigma Co. (St. Louis, MO, USA). All the procured chemicals were analytical grade.

DGG-9053A electric blast drying oven (Shanghai Senxin Experimental Instrument Co., Shanghai, China); MJ-10A pulverizer (Shanghai Puheng Information Technology Co., Shanghai, China); RVA 4500 Rapid Visco Analyzer (Perten Instruments, Hägersten, Sweden); scanning electron microscope (FEI, Lausanne, Switzerland); gel chromatography-differential-multiangular laser light scatter and Waters1525 high-performance gas chromatograph by US Waters (Shanghai, China), UV Spectrophotometer by General Analysis General Instruments Co., Ltd. (Beijing, China).

### Sample Preparation

#### Naturally Fermented Corn

The whole corn ballast (150 g) was weighed, washed three times with distilled water, added to distilled water (300 g), and naturally fermented at 30 °C for 1-20 d.

#### Determination of Chemical Constituents in Naturally Fermented Corn Ballast Powder

##### Determination of Crude Fat Content

Fill a filter article tube with 2 g of corn ballast powder that has been naturally fermented for 1–20 days and place it in the extractor. Connect the extractor and a dry extraction bottle with a known constant weight to the condenser, add 50 mL petroleum ether from the top of the condenser, connect the condensed water, start distillation at about 65°C, the number of siphons per hour is around 20. The filter article cylinder was removed after 6 h of distillation, the petroleum ether was recovered, and the extraction bottle was weighed after 1 h of drying in a drying oven at 105°C.

##### Determination of Crude Protein Content

Determine the crude protein content of 1 g of corn ballast powder that has been naturally fermented for 1–20 days using the Kjeldahl method.

##### Determination of Ash

Weigh 2 g of corn ballast powder that has been naturally fermented for 1–20 days in a crucible of known constant weight, stagger the lid by about 0.5 cm, and place it on an electric furnace to burn and carbonize until it is smokeless, then place it in a muffle furnace to burn at 550 °C for 23 h until the sample turns white or off-white, cool, and weigh. Then burn for another 0.5 h, remove, cool, and weigh until the difference in mass between the two times is <0.2 mg, which is the constant weight.

#### Extraction of Fermented Corn Starch

Corn ballast fermented for different times was rinsed with water, ground, and dried at 40 °C; it was then pulverized and passed through an 80-mesh sieve. The corn flour was extracted in 1:3 g/mL NaOH solution (0.3 g/100 mL) for 3 h, then centrifuged at 4,000 r/min for 10 min. The supernatant was discarded, and the upper layer of yellow-brown material in the precipitate was removed. The remaining material was then rinsed with water four times and centrifuged until the starch slurry turned white. The starch slurry was adjusted to pH 7 with 1 mol/L HCl, centrifuged, dried at 30 °C, passed through an 80-mesh sieve, and set aside for future use (10).

### Chemical Composition of Carbohydrate

#### Determination of Amylose Content

##### Plotting the Standard Curve of Amylose

Amylose and amylopectin standards (0.1 g) were weighed and placed separately in a beaker, then anhydrous ethanol (1 mL) and NaOH (1.0 mol/L, 9 mL) were added, respectively. The contents were shaken well and heated in a boiling water bath for 10 min. After cooling, the solution was transferred to a 100 mL volumetric flask and filled to the mark to obtain 1 mg/mL amylose/amylopectin standard solution. The prepared amylose standard solution was added to five 100 mL volumetric flasks at various volumes of 0, 0.25, 0.5, 1, and 1.5 mL, followed by the addition of the prepared amylopectin standard solution at various volumes of 5, 4.75, 4.5, 4, and 3.5 mL, respectively. The blank contained 0.09 mol/L NaOH (5 mL), following the addition of 1 mL acetic acid solution (1 mol/mL) and 1 mL iodine reagent (2% potassium iodide and 0.2% iodine, m/v) in turn, and filled up to 25 mL with water. The color was developed for 10 min and measured at 620 nm with a calorimeter. The absorbance values were recorded. The absorbance value was recorded at 620 nm. The amylose standard curve was plotted with the mass of amylose as the x-coordinate and the absorbance value as the y-coordinate.

##### Effect of Natural Fermentation on the Amylose Content in Corn Ballast

The starch sample (0.1 g) was weighed after different fermentation times and was prepared using the same procedures detailed in the previous section. The solution was cooled and filled to the mark in a 100-mL volumetric flask. Acetic acid solution (1 mL) and iodine reagent (1 mL) were added and water was added to fill to the 50-mL mark. After 10 min of color development, the absorbance was measured at 620 nm.

### Functional Properties

#### Effect of Natural Fermentation on the Solubility and Swelling Power of Corn Ballast Starch

The solubility and swelling power of corn ballast starch with different fermentation times were determined using the method proposed by Lv et al. (11). The 2% starch solution (50 mL) was prepared by adding fermented starch (1 g). It was placed in a boiling water bath for 30 min and shaken every 5 min. It was then cooled and centrifuged at 3,000 rpm for 15 min. The supernatant and precipitate were separated; the supernatant was oven-dried and the pellet was directly weighed.

Solubility index (SI) = A/W × 100%

Swelling power (SP) =P/W × (1-S)

where, W is the sample mass (g), A is the dry weight of the supernatant (g), and P is the weight of the pellet (g).

### Physical and Chemical Properties

#### Effect of Natural Fermentation on the Aging Properties of Corn Ballast Starch

Sample solutions were prepared in aluminum cylinders by adding 3.50 g (dry basis) fermented and unfermented corn ballast starch and 25 mL of distilled water. The device underwent the following controlled heating/cooling process: initial holding at 35 °C for 3 min, heating to 95 °C at a rate of 6 °C/min, holding for 5 min, and cooling to 50 °C at a rate of 6 °C/min. The result was plotted using the software provided by the device (12).

#### Effect of Natural Fermentation on the Pasting Properties of Corn Ballast Starch

Fermented corn ballast starch (3.0 mg, dry basis) and distilled water (7 μL) were added to the crucible, which was then repeatedly pressed three to four times with a sealing press until the edge of the crucible was properly sealed. The samples were equilibrated at room temperature for 12 h and their pasting characteristic curves were determined under the following conditions: N_2_ flow rate of 150 mL/min, the pressure of 0.1 MPa, and a temperature ramping rate of 5 °C/min (13).

### Structural Characterization

#### Effect of Natural Fermentation on the Molecular Weight of Corn Ballast Starch

Sample processing: 1 mL of 90% DMSO was added to 10 mg of the starch sample and the mixture was heated at 100 °C overnight, followed by the addition of 3 mL of anhydrous ethanol. After the solution was centrifuged, the supernatant was removed and the precipitate was rinsed twice with anhydrous ethanol and air-dried. Subsequently, 3 mL of 1 mol/L NaNO_3_ (containing 0.02% NaN_3_) was added to the dried sample, and the mixture was reacted at 121 °C for 20 min, then centrifuged at 12,000 r/min for 10 min. The prepared sample (100 μL) was analyzed using ASTRA 6.1 software (14).

Instrumentation analysis settings: the mobile phase was 0.1 mol/L NaNO_3_ (containing 0.02% NaN_3_), the flow rate was 0.4 mL/min, the column temperature was 60 °C, the analytical columns were Ohpak SB-804 HQ and Ohpak SB-806 HQ, and the loading volume was 100 μL.

#### Effect of Natural Fermentation on the Chain Length Distribution of Maize Ballast Starch

Purified starch (5 mg) was weighed, resuspended in water (0.9 mL), heated in a boiling water bath for 15 min under intermittent vortex mixing. The solution was then mixed with 0.1 mL of sodium acetate (0.1 M, pH 3.5), NaN_3_ (5 mL, 40 mg/mL), and 2.5 mL isoamylase, and heated in a water bath at 37 °C for 3 h. Afterward, 5 mL of anhydrous ethanol was added to the solution, followed by centrifugation at 4,000 rpm for 10 min. A DMSO/LiBr solution (1 mL) was added and dissolved by heating at 80 °C for 2 h.

The chromatographic device was a GPC-MALS system, consisting of a U3000 liquid-phase system (Thermo, USA), an Optilab T-rEX differential detector (Wyatt technology, CA, USA), and a DAWN HELEOS-II laser light scattering detector (Wyatt Technology, CA, USA).

According to the properties of specific compounds, gel exclusion columns (Ohpak SB-805 HQ: 300 × 8 mm, Ohpak SB-803 HQ: 300 × 8 mm) with appropriate molecular weight ranges were used, the column temperature was 60 °C, injection volume was 100 μL, mobile phase A was 0.5% LiBr and DMSO, the flow rate was 0.3 mL/min, and the gradient and isocratic elution for 120 min.

#### Effect of Natural Fermentation on the Crystallinity of Corn Starch

##### X-Ray Diffractometer Analysis Parameters

Conditions: CuKa radiation; power: 1,600 W; tube current: 40 mV; tube voltage: 4.0 × 10^4^ V; scanning speed: 4°/min; scanning range (2θ): 3–60°; step size: 0.02°; DS-SS-RS settings: 1 mm-1 mm-0.1 mm, respectively (15). The crystallinity was calculated using X'pert HighScore software and the corresponding curves were derived.

#### Effect of Natural Fermentation on the Surface Morphology of Corn Ballast Starch Granules

Homogeneously dispersed samples of fermented corn ballast starch were fixed with electrically conductive adhesive, coated with gold using an ion sputter coater, observed using SEM, and representative photographs were taken.

### Statistical Analysis

Microsoft Excel (Redmond, WA, USA) and SPSS software (IBM Corp, Armonk, NY, USA) were used for statistical analysis. Origin software (OriginLab Corporation, Northampton, MA, USA) was used for image processing. All data were collected in triplicate and the average value was used for analysis.

## Results

### Effects of Natural Fermentation on the Content of Chemical Constituents of Corn Ballast

Table 1 shows the impact of different fermentation times on the content of basic chemical components in corn ballast. The results of the experiments show that as the fermentation time is extended, the crude fat, crude protein, and ash content of corn ballast decreases.

**Table 1:** Effects of natural fermentation on the content of chemical constituents of corn ballast.

**Fermentation time/d**	**1**	**3**	**5**	**10**	**15**	**20**
Protein (%)	8.52 ± 0.11 a	8.11 ± 0.05 b	8.18 ± 0.06 b	7.94 ± 0.09 c	7.79 ± 0.12 c	7.62 ± 0.08 d
Fat (%)	4.21 ± 0.04 b	4.41 ± 0.03 a	4.05 ± 0.04 c	3.88 ± 0.05 d	3.92 ± 0.03 d	3.74 ± 0.06 e
Ash (%)	1.52 ± 0.02 a	1.50 ± 0.03 ab	1.45 ± 0.05 b	1.38 ± 0.02 c	1.37 ± 0.04 c	1.31 ± 0.03 d

*Equal letters in the rows do not differ in the Student's t-test with a significance level p < 0.05*.

### Effect of Fermentation on the Amylose Content in Corn Ballast Starch

#### Plotting the Standard Curve for Amylose

**Figure 1:**
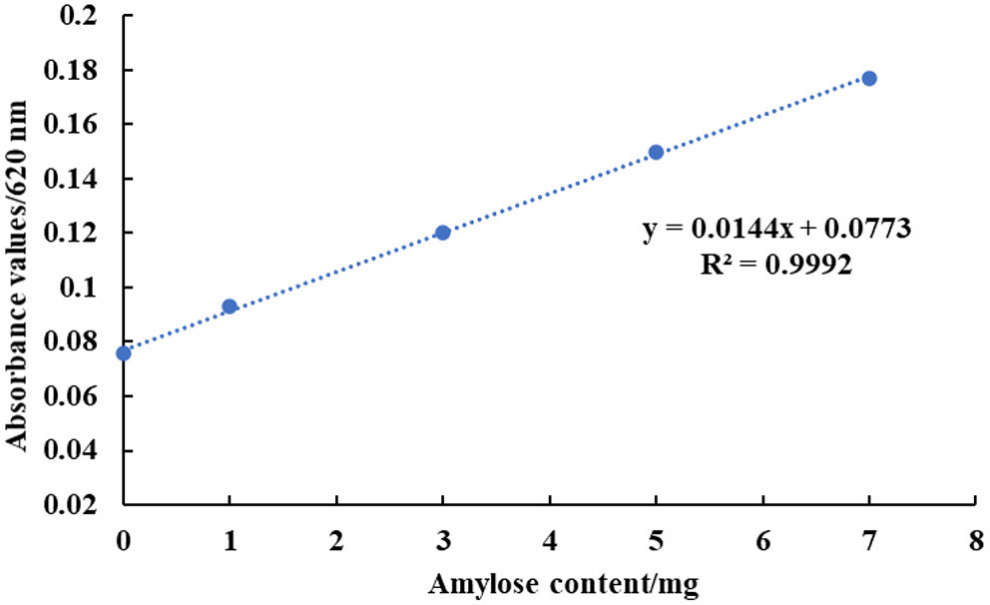
Standard curve of amylose.

The absorbance value of the solution was measured at a wavelength of 620 nm and the standard curve was plotted with the mass of amylose as the x-coordinate and the absorbance value as the y-coordinate. The results were as shown in Figure 1: y = 0.0144x + 0.0773, where the coefficient of determination is R^2^ = 0.9992, indicating a good linear relationship between the amylose content and the absorbance value.

#### Effect of Natural Fermentation on the Amylose Content in Corn Ballast

**Figure 2:**
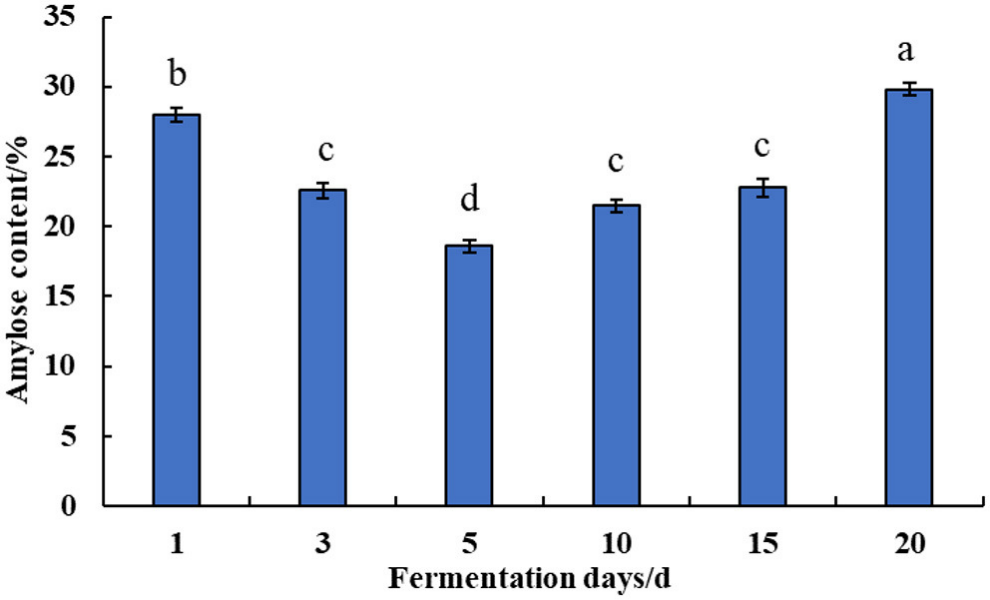
Effect of natural fermentation on the content of amylose in corn ballast. The same letters in the figure do not differ in the student's *t*-test with a significance level *p* < 0.05.

The effect of natural fermentation on the content of amylose in corn ballast is shown in Figure 2. The corn was subjected to natural fermentation for 1–20 d. The amylose content in corn ballast starch decreased slightly from 27.98 to 18.61% in the first 1–5 d of fermentation, then it gradually increased in the later stage of fermentation as fermentation proceeded, reaching significantly higher levels relative to the content in the early stage of fermentation.

### Effect of Natural Fermentation on the Functional Properties of Maize Ballast Starch

#### Effect of Natural Fermentation on the Solubility and Swelling Power (SP) and Solubility Index (SI) of Corn Ballast Starch

The effect of natural fermentation on the solubility and swelling power of corn ballast is shown in Table 2. Corn ballast underwent natural fermentation for 120 d, during which its swelling power indicator showed an initial increase followed by a subsequent decrease as fermentation proceeded, with the highest swelling value of 12.18 g/g appearing on day 5. On the contrary, the starch solubility showed the opposite trend, that is, the solubility decreased at the beginning of fermentation, then increased during the later stage of fermentation.

**Table 2:** Effect of natural fermentation on the solubility and swelling power of corn ballast starch.

**Fermentation time/d**	**1**	**3**	**5**	**10**	**15**	**20**
Swelling power (SP) (g/g)	8.02 ± 0.08 d	11.23 ± 0.15 b	12.18 ± 0.07 a	9.85 ± 0.11 c	9.81 ± 0.16 c	7.89 ± 0.17 d
solubility index (SI) (%)	14.2 ± 0.7 a	10.4 ± 0.3 b	2.3 ± 0.08 d	4.4 ± 0.18 c	10.2 ± 0.3b	10.1 ± 0.2 b

*Equal letters in the rows do not differ in the Student's t-test with a significance level p < 0.05*.

### Effect of Natural Fermentation on the Physicochemical Properties of Corn Ballast Starch

#### Effect of Natural Fermentation on the Pasting Properties of Corn Ballast Starch

Table 3 shows the pasting properties of corn ballast starch under different fermentation times. During the fermentation process, the peak viscosity, trough viscosity, viscosity breakdown, and final viscosity of starch uniformly showed a decreasing trend. When the pasting temperature increased, the thermal stability of starch paste increased, and the decrease in its setback viscosity indicated that the processibility and adaptability of corn ballast starch were improved by fermentation.

**Table 3:** Effect of natural fermentation on the pasting properties of corn ballast starch.

**Fermentation time/d**	**Parameters**						
	**PV** **(cP)**	**TV** **(cP)**	**BV** **(cP)**	**FV** **(cP)**	**SV** **(cP)**	**Peak time** **(min)**	**PT** **(°C)**
1	546 ± 24 a	309 ± 2 a	237 ± 5 b	483 ± 17 a	174 ± 15 a	4.27 ± 0.14*c*	76.5 ± 1.25 b
3	384 ± 18 b	308 ± 4a	75 ± 12e	336 ± 19 b	28 ± 15 b	5.53 ± 0.18 a	92.65 ± 2.02 a
5	601 ± 22 a	310 ± 3 a	291 ± 7 a	453 ± 22 a	143 ± 19 a	3.93 ± 0.09 d	74.9 ± 1.88 b
10	370 ± 19 b	281 ± 2 b	89 ± 5 de	325 ± 20 b	44 ± 18 b	5.27 ± 0.1 ab	90.35 ± 2.14 a
15	402 ± 19 b	296 ± 3 b	106 ± 6 cd	361 ± 25 b	65 ± 22 b	5.2 ± 0.05 b	90.2 ± 1.36 a
20	420 ± 24 b	296 ± 1 b	124 ± 10 c	339 ± 23 b	43 ± 22 b	5.27 ± 0.06 ab	90.15 ± 2.31 a

*Mean and standard deviation of three replicates. Equal letters in the columns do not differ in the Student's t-test with a significance level p < 0.05. PV, peak viscosity; TV, trough viscosity; BV, viscosity breakdown; FV, final viscosity; SV, setback viscosity; Peak time, time for maximum peak viscosity; PT, pasting temperature*.

#### Effect of Natural Fermentation on the Characteristic Parameters of Pasting in Corn Ballast Starch

The effect of natural fermentation on the pasting characteristics of corn ballast starch is shown in Table 4. During the 1–20 d fermentation process, the pasting temperature and pasting range of corn ballast starch showed an initial increase from 59.4 to 61.0 °C, followed by a subsequent decrease to 59.6 °C as fermentation progressed. The enthalpy value also showed the same trend, i.e., the enthalpy value of corn ballast starch increased from 8.651 to 9.120 J/g at the early stage of fermentation and gradually declined to 8.434 J/g as fermentation progressed.

**Table 4:** Effect of natural fermentation on the characteristic pasting parameters of corn ballast starch.

**Fermentation time/d**	**Parameters**				
	**To (°C)**	**Tp (°C)**	**Tc (°C)**	**ΔT (Tc–To)**	**ΔH (J/g)**
1	59.4 ± 0.38 d	67.3 ± 0.29 b	73.2 ± 0.28 d	13.8 ± 0.1 b	8.651 ± 0.71 a
3	60.6 ± 0.15 ab	67.2 ± 0.18 b	74.8 ± 0.31 b	14.2 ± 0.16 a	8.868 ± 0.55 a
5	61.0 ± 0.22 a	67.9 ± 0.16 a	75.3 ± 0.26 a	14.3 ± 0.04 a	9.120 ± 0.88 a
10	60.2 ± 0.25 bc	67.3 ± 0.28 b	74.0 ± 0.17 c	13.8 ± 0.08 b	9.071 ± 0.82 a
15	59.6 ± 0.36 cd	66.8 ± 0.22 c	73.4 ± 0.22 d	13.8 ± 0.14 b	8.662 ± 0.68 a
20	59.6 ± 0.14 cd	66.3 ± 0.05 c	73.2 ± 0.16 d	13.6 ± 0.02 c	8.434 ± 0.95 a

*Mean and standard deviation of three replicates. Equal letters in the columns do not differ in the Student's t-test with a significance level p < 0.05. To, starting pasting temperature; Tp, peak temperature; Tc, ending pasting temperature; ΔT, pasting range; ΔH, enthalpy values*.

### Effect of Natural Fermentation on the Molecular Structure of Corn Ballast Starch

#### Effect of Natural Fermentation on the Molecular Weight of Corn Ballast Starch

The effect of natural fermentation on the molecular weight of corn ballast starch is shown in Table 5. As fermentation progressed, the weight average molecular weight Mw, number average molecular weight Mn, and dispersion coefficient of fermented corn ballast starch uniformly showed an initial upward and subsequent downward trend. In particular, the weight average molecular weight increased from 7.13 × 10^7^ to 7.78 × 10^7^ g/mol in the early stage of fermentation, then gradually declined to 6.68 × 10^7^ g/mol as fermentation progressed. The high molecular weight (>4 × 10^8^ g/mol) first increased, then decreased as fermentation progressed. Meanwhile, the dispersion coefficient increased from 3.019 to 3.442, then decreased to 3.002. The dispersion coefficients of the starch molecules after fermentation were overall >1, indicating that the fermented starch molecules were more extensively dispersed and complex in structure.

**Table 5:** Effect of natural fermentation on the molecular weight of corn ballast starch.

**Fermentation time/d**	**Mw (g/mol)**	**Mn (g/mol)**	**Mw/Mn**	**Molecular weight distribution (%)**			
				**<1 × 10^8^** **(g/mol)**	**1 × 10^8^-2 × 10^8^** **(g/mol)**	**2 × 10^8^-4 × 10^8^** **(g/mol)**	**>4 × 10^8^** **(g/mol)**
1	7.13 × 10^7^	2.36 × 10^7^	3.019	83.6	9.4	6.0	1.0
3	7.78 × 10^7^	2.26 × 10^7^	3.442	82.9	9.4	6.1	1.6
5	7.61 × 10^7^	2.33 × 10^7^	3.267	82.6	9.8	5.8	1.8
10	7.45 × 10^7^	2.48 × 10^7^	3.002	83.4	9.2	6.1	1.3
15	6.68 × 10^7^	2.18 × 10^7^	3.059	84.5	9	5.7	0.8
20	672 × 10^7^	2.16 × 10^7^	3.112	84.6	8.6	6.1	0.7

#### Effect of Natural Fermentation on the Chain Length Distribution of Corn Ballast Starch

**Figure 3:**
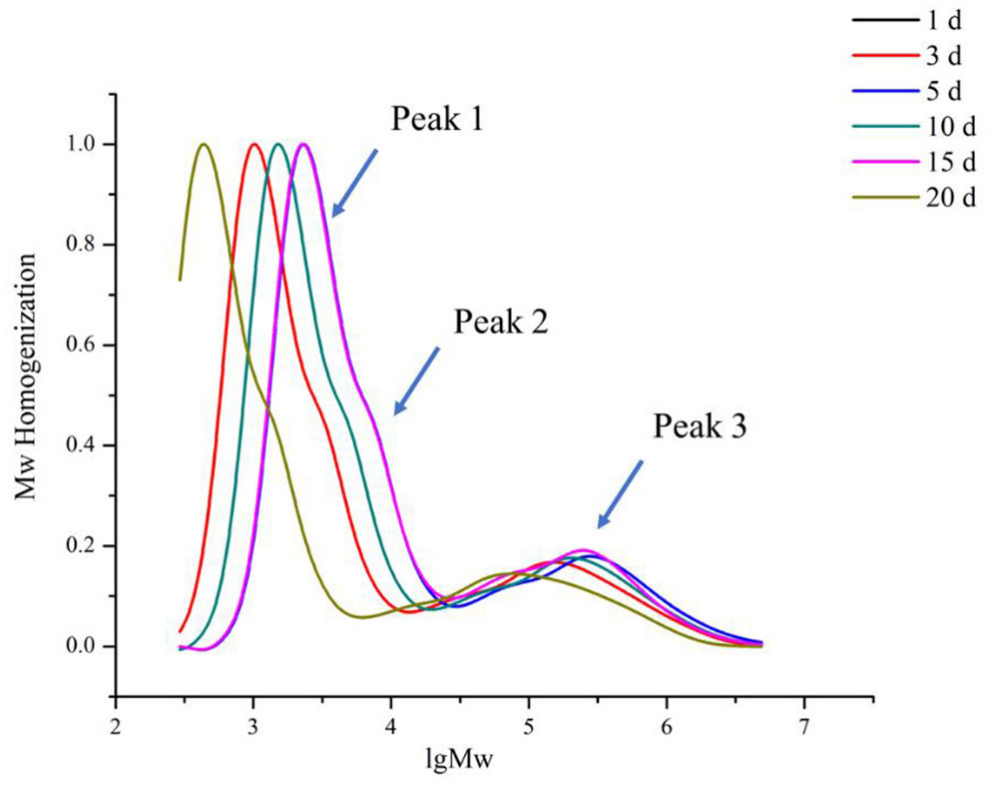
Effect of natural fermentation on the chain length distribution of corn ballast starch.

The effect of natural fermentation on the chain length distribution of corn ballast starch is shown in Figure 3 and Table 6. Peak 1 exhibited the largest molecular weight fraction of the fermented sample, and its relative content initially increased, then decreased as fermentation progressed, i.e., the content of large molecular weight fermented starch increased from 51.45 to 53.26% during short fermentation time, and decreased to 52.52% as fermentation continued. The small molecular weight portion of the fermented samples exhibited the opposite trend, i.e., the content decreased from 25.59 to 24.7%, then increased to 25.76%, which was consistent with the results of the molecular weight and molecular weight distribution of fermented corn ballast starch.

**Table 6:** Effect of natural fermentation on the chain length distribution of corn ballast starch.

**Fermentation time/d**	**Peak1_area**	**Peak_area (%)**	**Peak2_area**	**Peak2_area (%)**	**Peak3_area**	**Peak3_area (%)**
1	3.48	51.45	1.47	22.95	1.44	25.59
3	3.42	52.79	1.42	22.01	1.63	25.2
5	3.44	53.26	1.42	22.04	1.59	24.7
10	3.45	53.38	1.39	22.56	1.62	25.06
15	3.46	52.91	1.42	22.76	1.66	25.33
20	3.44	52.52	1.40	22.72	1.59	25.76

#### Effect of Natural Fermentation on the Crystallinity of Corn Ballast Starch

**Figure 4:**
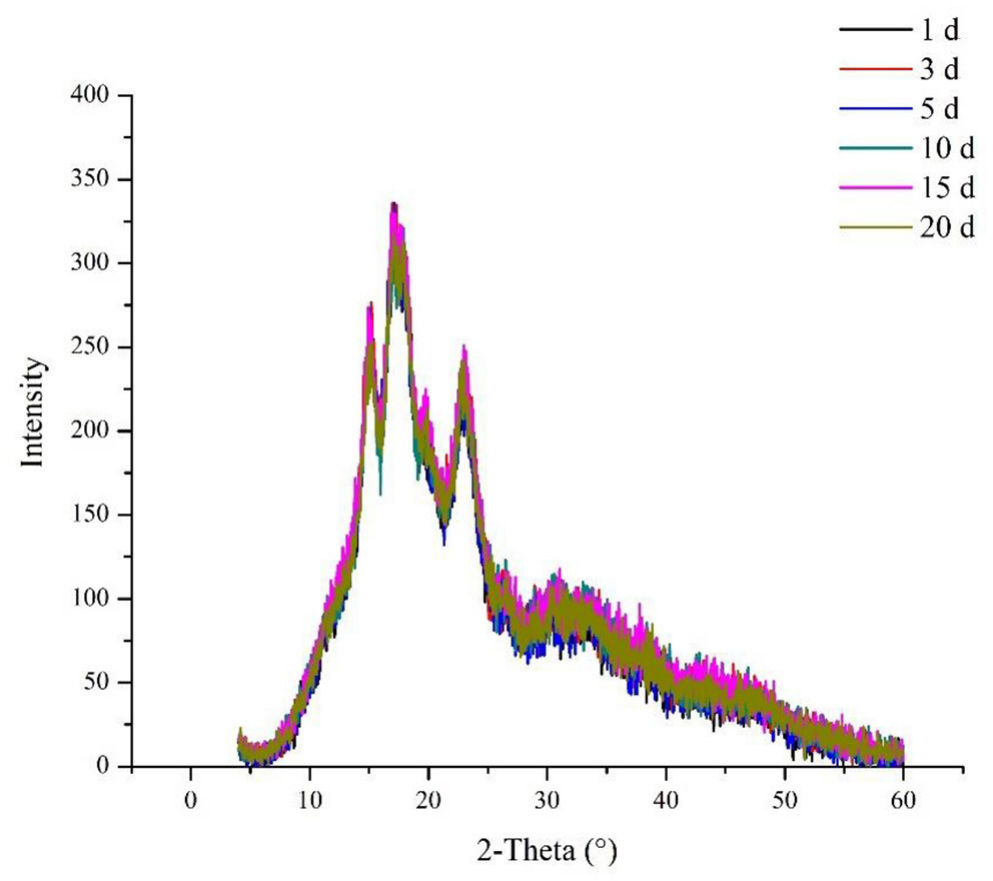
Effect of natural fermentation on the crystallinity of corn ballast starch.

The crystallinity of corn ballast starch with different fermentation times is shown in Figure 4 and Table 7. The fermented corn ballast starch revealed diffraction peaks at 15, 17, 18, 20, and 23°, which indicated that the crystalline structure of starch (a typical A-type structure) remained the same at different fermentation times. Although the crystalline structure of corn starch sampled did not vary with different fermentation times, the intensity of diffraction peaks varied. The crystallinity of fermented starch displayed an initial increase and subsequent decrease as the fermentation time extended. The crystallinity of ballast starch increased from 10.14 to 22.09% under short-term fermentation and gradually decreased from 22.09 to 19.09% as fermentation progressed.

**Table 7:** Effect of natural fermentation on the crystallinity of corn ballast starch.

**Fermentation time/d**	**Diffraction angle 2θ (°)**					**Degree of crystallinity (%)**	**Crystal pattern**
	**15°**	**17°**	**18°**	**20°**	**23°**		
1	2.2	4.9	1.1	0.4	2.7	10.14	A
3	6.8	5.3	5.8	3.1	5.2	20.86	A
5	6.9	5.7	7.3	3.8	4.6	22.09	A
10	7.9	7.7	2.7	3.4	6.3	21.86	A
15	7.3	8.6	2.6	3.0	5.6	21.37	A
20	4.7	6.1	5.4	3.4	4.5	19.09	A

#### Effect of Natural Fermentation on the Surface Morphology of Corn Ballast Starch Granules

**Figure 5:**
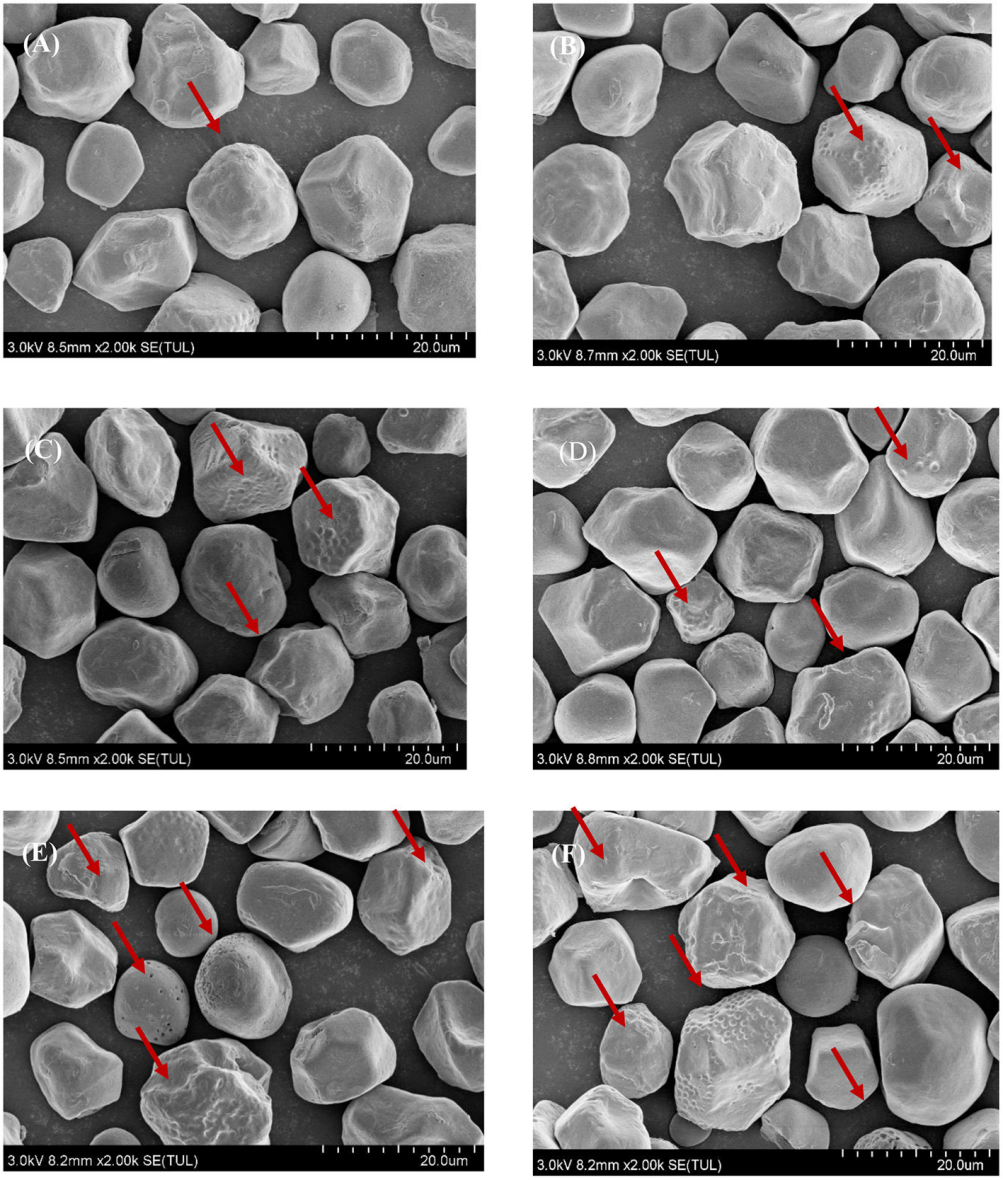
Effect of natural fermentation on the surface morphology of corn ballast starch granules. **(A)** Fermentation time:1 d; **(B)** Fermentation time:3 d; **(C)** Fermentation time:5 d; **(D)** Fermentation time:10 d; **(E)** Fermentation time:15 d; **(F)** Fermentation time:20 d.

The effect of natural fermentation on the surface morphology of corn ballast starch granules is shown in Figure 5. The granules had various sizes in irregular polygon shapes. The surface of the granules was relatively rough and cavities were formed inside the granules. As fermentation progressed, more pores and cracks appeared on the surface of the granules, with granule deformation and breakdown observed occasionally.

## Discussion

As the primary nutrient component of corn and other food crops, starch has unique functional properties such as swelling power, pasting parameters, and regeneration value. The functional properties of starch are mainly determined by the ratio of amylopectin and amylose in the starch. The amylopectin content is positively correlated with starch swelling and amylose tends to undergo aging and polymerization processes (16). Therefore, corn ballast was naturally fermented for 1–20 d. The amylose content of corn ballast was measured in triplicate, and the average was determined, as shown in Figure 2. As fermentation progressed, the amylose in corn ballast starch showed a downward trend, followed by an upward trend. The initial decrease in amylose during early fermentation might be due to the erosion of starch granules by lactic acid produced by microbial metabolism during the natural fermentation process, which caused the formation of openings on its surface and the outflow of amylose. The organic acids or enzymes produced by the metabolism of the dominant microorganisms during fermentation worked on the amorphous zone, a relatively loose structure of starch, and weakened or even destroyed the amorphous zone, i.e., the non-crystalline zone, thus resulting in the dissolution of amylose. The α-amylase produced by microbial metabolism degraded the amylose into small molecules such as dextrin or monosaccharides, which then escaped from the starch granules (17). Therefore, the amylose content decreased within a short period of natural fermentation. This was similar to the findings of Bian et al. (18). As fermentation progressed, amylases such as β-amylase and organic acids produced by the microbial metabolism of natural fermentation caused a certain degree of degradation and debranching of amylopectin to produce amylose of small molecular weight. As fermentation proceeded, the fat and protein ash in corn underwent a certain degree of degradation to purify the amylose molecules, so its relative content increases after fermentation.

Studies have shown that under the conditions of starch milk formation, heating led to the breakage of chemical bonds between starch molecules, thus forming new hydration sites and promoting the swelling of starch granules. Since amylopectin molecules have a three-dimensional branching structure containing more spatial sites to bond to more water molecules, the amylopectin content is positively correlated with the change in swelling index (19, 20). The determination of amylose content showed that the swelling power increased at the beginning of fermentation when the amylose content decreased accompanied with the increased amylopectin content (Table 2). As fermentation progressed, the starch molecules were hydrolyzed into small molecules such as dextrin and oligosaccharides in the presence of organic acids and amylase, causing the swelling power to decrease significantly. The increase in amylose content at the later stage of fermentation further inhibited the absorption of water and swelling of starch because, under heating conditions, the lipids and amylose were more likely to form water-insoluble complexes. The trend of water solubility of corn starch after fermentation was opposite to that of the swelling power, showing an initial decrease, followed by a subsequent increase. The results of this analysis were consistent with the results of amylose content, with the rebounding solubility indicating more amylose was dissolved in the later stage of fermentation (21).

The aging characteristic curves obtained from RVA are closely related to the quality of starchy foods and are also important parameters for evaluating the quality of cooking characteristics of food crops. In this experiment, the aging characteristic values of corn ballast starch with different fermentation times were determined, and the results are shown in Table 3. During fermentation, the peak viscosity of starch showed a decreasing trend, and the variation in the viscosity characteristic values of starch in the later stage of fermentation was not significant. As an indirect indicator of starch hydration, viscosity showed a positive correlation with the branching degree of amylopectin molecules, that is, the higher the branching degree, the higher the ability of starch molecules to absorb and retain water molecules, and the greater the viscosity. In this study, during extended fermentation, the molecular weight of starch was reduced, and the branched starch degraded, which changed their molecular structure and limited the hydration of starch granules, therefore resulting in declining swelling force and viscosity. Studies by Camargo et al. (22), Chang et al. (23), and Han et al. (24) revealed that the long-chain content of amylopectin had a high positive correlation with the attenuation value. During the later stage of fermentation, the percentage of macromolecules in starch decreased. Hence, fermentation lowered the attenuation value and the starch granules became more susceptible to swelling, leading to higher stability (25). The starch pasting temperature after fermentation showed an increasing trend, mainly because of the presence of low molecular substances, i.e., dextrin and sugar, which bonded to water molecules and inhibited the hydration and swelling of starch, thus increasing the pasting temperature. Meanwhile, the production of microbial metabolites such as organic acids during the fermentation process destroyed the crystalline region of the starch molecules. The content of amylose and short amylopectin increased, and the crystallinity was enhanced, so higher temperature was required to destroy the crystalline region of starch, thus reducing the aging and pasting of starch and the regeneration value.

The variation trends of the starting pasting temperature (To), peak temperature (Tp), ending pasting temperature (Tc), pasting range (ΔT), and the enthalpy values of corn ballast starch for different fermentation times are shown in Table 4. The results indicated that the pasting temperature range of corn ballast starch showed an initial increasing trend followed by a decrease, which was caused by the uneven surface of corn ballast starch granules during fermentation. Meanwhile, the increase in enthalpy value from 8.651 to 9.120 J/g was due to the destruction of the non-crystalline region of the starch granules in the early stage of fermentation and the increase in crystallinity, which increased in the relative proportion of structurally stable crystalline regions. Hence, a higher pasting temperature was required to disrupt the crystalline regions of the starch and the enthalpy required for starch gelation also increased (26). The pasting temperature is a common characteristic parameter of starch and refers to the temperature at which the starch granules change from the crystalline state to the gel under heating conditions. It first increased, then decreased as fermentation proceeded. The pasting temperature range also followed the same trend. In the early stage of fermentation, the amylose content decreased while the amylopectin content increased, thus making it more difficult for starch molecules to absorb water and swell, thus delaying the pasting of starch molecules (27). In the later stage of fermentation, the pasting temperature and pasting temperature range of corn starch gradually decreased, mainly because the amylose content slowly increased, the starch crystallinity decreased, and the starch molecular weight decreased as fermentation proceeded.

As a homologous mixture of macromolecular compounds, the molecular weight of starch directly affects its molecular structure and physicochemical properties, such as aging and pasting properties (28). The effects of different fermentation times on the weight average molecular weight Mw and number average molecular weight Mn of corn ballast starch are shown in Table 5. The short fermentation time increased the molecular weight of corn ballast starch, where the low molecular weight content (<1 × 10^8^ g/mol) decreased from 83.6 to 82.6%, and the high molecular weight content (Mw>4 × 10^8^ g/mol) increased from 1 to 1.8%. During early fermentation, because the amylose in the corn granules was completely dissolved, microorganisms adequately hydrolyzed amylose to produce sugars of small molecular weight, which were reused as a carbon source by microorganisms in the fermentation broth, resulting in a decrease in the relative content of amylose and a decline in the low molecular weight content. Meanwhile, the hydrolysis of amylose caused an increase in the relative content of amylopectin and an increase in molecular weight. As fermentation progressed, the microbial flora structure became more diverse and produced considerable amounts of amylase, glycosylase, and organic acids, which facilitated hydrolysis of the α-1,4 and α-1,6 bonds in starch to generate glucose and decreased the molar mass (29). When the ratio of Mw/Mn was >1, a broader range of molecular distribution or larger starch granules was observed. The experimental results showed that after fermentation, the dispersion coefficients were uniformly >1, indicating that the fermentation resulted in uneven distribution of the starch molecules. The decreased value was mainly due to the degradation of certain short amylopectin and amylose into sugars of low molecular weight, which was consistent with the change in amylose content.

GPC elutes the molecules in the solvent in order of size, with larger molecules entering the gel particles with larger pore size, thus shortening their travel distance within the gel bed, whereas the smaller molecules moved for a longer distance (30). Hence, molecules with high molecular weights were eluted first to form peak 1, whereas small molecules were eluted last as peak 3. Figure 3 and Table 6 show the starch chain length distribution of the fermentation samples. The corn starch of high molecular weight mainly contained amylopectin, shown in peak 1. The relative content of large molecules increased in the early fermentation stage, because the organic acids and other substances accumulated in the fermentation broth formed holes on the starch granules, so amylose leaked out, then was hydrolyzed by amylase and other substances. Thus, the amylose content of starch in the early stage of fermentation decreased, and the relative content of amylopectin increased, resulting in increased molecular weight and macromolecule content. As fermentation progressed, the percentage of macromolecules in peak 1 decreased from 53.38 to 52.52%, whereas the percentage of small molecules in peak 3 increased from 25.06 to 25.76%, possibly because long amylopectin mainly existed in the crystalline region of the starch molecules, and the α-amylase and β-amylase produced during fermentation hydrolyzed the amorphous region of starch, thus causing debranching of the long amylopectin molecules to form medium and short amylose molecules. This resulted in a decrease in the content of large molecules and an increase in the content of small molecules (31).

Figure 4 and Table 7 show the X-ray diffraction patterns of corn ballast starch at different fermentation times. The results indicated that the fermented corn ballast starch had a typical A-type crystalline structure, with diffraction peaks at 15, 17, 18, 20, and 23°. Although the peak height changed to some extent, the crystalline structure of the starch did not change but remained as the A-type for all fermented samples. The presence of absorption peaks at 2θ = 23° in the diffraction pattern indicated the presence of amylopectin (32). The intensity of the absorption peaks first increased, then decreased, indicating that the amylopectin content in the fermentation samples increased and then decreased, which correlated with the results of amylose content, variation of molecular weight, and chain length distribution. The amorphous region of the starch granules consists primarily of amylose and amylopectin in disordered conformation and branching points, whereas the crystalline region contains crystalline structures formed by side chains of amylopectin molecules in a double helix form (33). At the beginning of fermentation, the amylose molecules in the amorphous region were hydrolyzed by amylase and organic acids produced by microbial metabolism. The amorphous region was destroyed, and the crystallinity increased from 10.14 to 22.09%, which in turn hindered starch pasting and increased the pasting temperature. This result correlated with the results of the DSC experiment. As fermentation progressed, the crystallinity decreased significantly. As fermentation progressed, the crystalline region of corn starch underwent hydrolysis, and the short amylopectin on the edge of the crystalline region was degraded to produce amylose, so the content of small molecules in the later stage of fermentation increased, leading to a reduction in the relative proportion of the crystalline region of starch, i.e., the crystallinity of starch was reduced by fermentation.

SEM analysis enables direct observation of the microscopic morphology of corn ballast starch at different fermentation times, thus explaining the effect of different fermentation times on the physicochemical and functional properties. SEM results are shown in Figure 5. During natural fermentation, corn ballast starch was hydrolyzed by microbial metabolites, resulting in a decrease in particle size and irregular shapes, which in turn changed the water absorption of corn ballast starch, resulting in various solubility and swelling forces. The appearance of noticeable holes on the surface of corn ballast starch granules at different fermentation periods confirmed that fermentation damaged the starch granules. As the fermentation time increased, erosion and cracks occurred more extensively on the granule surface, and small holes appeared, leading to the formation of porous network structures. The experimental results correlated with those of Reyes et al. (17). Research by Lu et al. (34) showed that the ratio of amylose to amylopectin was directly related to the increase in pores and the change in granule surface, and this study further confirmed the changes in amylose content and molecular weight during the natural fermentation process.

## Conclusions

We studied the effects of different fermentation times on the physicochemical indicators, functional properties, and molecular structures of corn ballast starch. The results will provide important guidance in the application of fermentation technology to improve the quality of traditional corn ballast foods. In this study, corn ballast was naturally fermented for 1–20 d, and the molecular structure and physicochemical characterization of corn ballast starch under different fermentation times were explored. The results showed that the amylose content and starch solubility first decreased, then increased with the increase in fermentation time, while the swelling power displayed the opposite trend. SEM results indicated that the deformation of starch granules worsened as fermentation progressed. The viscosity of starch showed a negative correlation with the fermentation time. The molecular weight increased, then decreased as the fermentation time progressed. The results of chain length distribution further confirmed that short-term fermentation increased the content of large molecules of corn ballast starch, which then decreased as the fermentation time progressed. However, different fermentation times did not affect the crystalline structure of the starch. The crystallinity initially grew then declined as the fermentation time changed, which in turn resulted in an initial increase and subsequent decrease in the pasting temperature. During short-term fermentation, the retrogradation value of corn ballast starch is low, resulting in fermented starch products with a soft gel texture and low elasticity, making them suited for anti-aging products such as steamed bread and bread. When the fermentation time exceeds 10 days, the gelatinization temperature and enthalpy value fall as the amylose concentration rises, which is appropriate for the development of aging products like vermicelli. Thus, the experimental results demonstrated the potential of fermentation technology in improving the quality of corn ballast starch food. However, more research and development is needed to understand the influence of natural fermentation on the molecular structure and physicochemical properties of corn ballast starch, such as understanding the structure of the microbial flora and metabolites in the fermentation broth. As a result, the later works of the manuscript mostly focus on the use of metagenomics and metabolomics to further investigate the impact of bacterial flora and metabolites on starch molecular structure.

## Data Availability Statement

The original contributions presented in the study are included in the article/supplementary material, further inquiries can be directed to the corresponding author.

## Author Contributions

CW and YG were responsible for the design, overall management of the entire study, and editing. SZ, DL, and JJ provided the validation and formal analysis. YWu, XH, and MW: writing-review and editing. YWa, WW, and LW analyzed the data. LC: supervision, writing-review and editing, and funding acquisition. All authors have read and agreed to the publishing of the current version of the manuscript.

## Funding

This study was funded by the National Key Research and Development Plan (grant No. 2017YFD0401203), the Heilongjiang Provincial Agricultural Reclamation Bureau Science and Technology Project (grant No. HNK135-05-02), Heilongjiang Provincial Natural Science Foundation of China (LH2020C087), and the Heilongjiang Bayi Agricultural University Graduate Innovation Research Project (grant No. YJSCX2018-Y53).

## Conflict of Interest

The authors declare that the research was conducted in the absence of any commercial or financial relationships that could be construed as a potential conflict of interest.

## Publisher's Note

All claims expressed in this article are solely those of the authors and do not necessarily represent those of their affiliated organizations, or those of the publisher, the editors and the reviewers. Any product that may be evaluated in this article, or claim that may be made by its manufacturer, is not guaranteed or endorsed by the publisher.
